# Past, Present, and Future of Nerve Conduits in the Treatment of Peripheral Nerve Injury

**DOI:** 10.1155/2015/237507

**Published:** 2015-09-27

**Authors:** Aikeremujiang Muheremu, Qiang Ao

**Affiliations:** ^1^Medical Center, Tsinghua University, Beijing 8610084, China; ^2^Department of Orthopedics, Fifth Affiliated Hospital of Xinjiang Medical University, Urumqi, Xinjiang 86838010, China; ^3^Institute of Tissue Engineering, China Medical University, No. 77 Puhe Road, Shenyang North New Area, Shenyang, Liaoning 86110122, China

## Abstract

With significant advances in the research and application of nerve conduits, they have been used to repair peripheral nerve injury for several decades. Nerve conduits range from biological tubes to synthetic tubes, and from nondegradable tubes to biodegradable tubes. Researchers have explored hollow tubes, tubes filled with scaffolds containing neurotrophic factors, and those seeded with Schwann cells or stem cells. The therapeutic effect of nerve conduits is improving with increasing choice of conduit material, new construction of conduits, and the inclusion of neurotrophic factors and support cells in the conduits. Improvements in functional outcomes are expected when these are optimized for use in clinical practice.

## 1. Introduction

Peripheral nerve injuries, which affect 13 to 23 per 100000 persons each year, are one of the main problems in level one trauma centers [1, 2]. Since most of the patients with peripheral nerve injury are at the peak of their employment productivity, any loss or decrease in function can be particularly devastating [3]. Treatment of injuries to peripheral nerves is one of the most challenging surgical problems. Despite advancements in microsurgical techniques, complete recovery of nerve function after repair has never been achieved [4]. The results of peripheral nerve repair have reached a plateau, with functional recovery still being unsatisfactory, and surgical techniques can hardly be further refined.

Despite early diagnosis and accurate nerve repair with modern surgical techniques, functional recovery never reached the preinjury level. Poor outcomes result from factors both intrinsic and extrinsic to the nervous system, such as the type and level of injury, integrity of the surrounding tissues, the timing of the surgery, and changes in spinal cord neurons and end organs [5–7]. Misdirection of regenerating axons at the injury site is still a major problem. Therefore, interest is increasing in the role of microenvironmental factors in regulating accurate axonal regeneration.

Different from the central nervous system, the peripheral nervous system has strong potential for regeneration. Within an appropriate microenvironment, the regenerating axons extend their processes into the distal bands of Bunger to restore the function of end organs. Traditional epineurium neurorrhaphy for peripheral nerve injury induces regeneration by direct contact, which leads to enforced inosculation and inappropriate coaptation of nerve fascicles, which may result in neuroma. Nerve grafting remains the gold standard of care in addressing peripheral nerve injuries that cannot be bridged by direct epineural suturing [8]. However, the autologous nerve graft is very limited and not readily available; the process of harvesting autologous nerve graft results in morbidity, scarring, sensory loss, and neuroma formation at the site of harvest [9–11]. Thus, it is necessary to take a different approach than direct neurorrhaphy and nerve grafting to achieve satisfactory functional recovery with little complications, particularly in patients with extensive peripheral nerve injury and insufficient amount of donor nerve for harvest.

## 2. An Alternative to Nerve Autograft

**Figure 1:**
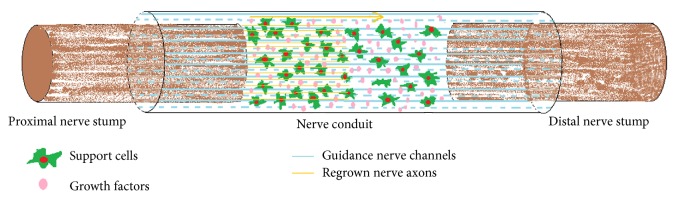
The preferable microenvironment created by the nerve conduit that promotes selective nerve regeneration.

Application of nerve conduits can effectively solve the problems of direct nerve suturing and nerve grafting. In nerve conduit bridging technique, proximal and distal nerve stumps would be inserted into the two ends of a nerve conduit, and axons regenerating from the proximal stump grow through the conduit and selectively grew into their original pathways in the distal stump. The conduit provides trophic support for both stumps and prevents the invasion of the surrounding tissues into the gap between two stumps. Moreover, nerve conduits enrich the neurotrophic factors within the chamber and build a microenvironment, which enhances axonal regeneration after injury (Figure 1).

Since Cajal [12] proposed the hypothesis of nerve chemotaxis, nerve conduit bridging has been developed and gradually used in the clinic. Brushart et al. [13], in their study grafting rat sciatic nerves with nerve conduits, found that the microenvironment produced by nerve conduit is beneficial for robust and accurate nerve regeneration and functional recovery. In other series of studies, Koerber et al. [14] reported better recovery using nerve conduits than direct nerve suturing in animal subjects. Meek and Coert [15] persuaded the EU and US Food and Drug Administration to test the effectiveness of various absorbable nerve conduits. Weber et al. [16] performed a randomized controlled study of 136 patients with peripheral nerve injury in 5 US medical centers. Patients were randomly distributed to polyglycolic acid (PGA) bridging or direct suturing groups: 91% of the PGA group reported satisfactory healing results as compared with 49% of the direct suturing group. Taras et al. [17] performed peripheral nerve conduit repair in 73 patients with peripheral nerve injuries, and, except for 2 patients with allergy, all the other patients reported satisfactory recovery. Ashley et al. [18] used a NeuraGen conduit to treat 7 infants with obstetric brachial plexus palsy. All infants gained satisfactory nerve functions and did not suffer from any complications.

Summarizing from the current animal studies and clinical trials, artificial nerve conduit grafting method is superior to direct suturing and autologous nerve grafting in that conduits can be easily prepared, can be shaped into any size, can be readily available in the surgery, can prevent the functional loss at the donor site, and can promote the axonal regeneration accuracy after nerve injury.

## 3. Desirable Properties of Nerve Guidance Conduits

In order to provide ideal scaffold and channel for axonal regeneration, the materials of the nerve guidance conduits should have the following physical properties [19–21].

### 3.1. Permeability

Nutrients and oxygen need to diffuse into the site of regeneration before the tube becomes vascularized. In addition, permeability might be needed to ensure viability of supportive cells if added.

### 3.2. Flexibility

Nerve conduits should be flexible to avoid causing mechanical injury to the surrounding tissues and regenerating axons. It is especially important when the nerve conduit is sutured over a joint.

### 3.3. Swelling

Inappropriate swelling could block the tunnel and prevent nerve regeneration through the conduit or directly injure the regenerated nerve in the conduit.

### 3.4. Rate of Degradation

The ideal nerve conduit should remain intact before the axons grow from the proximal stump through the gap to reinnervate the distal nerve pathways and then degrade gradually with minimal swelling or the surrounding tissues [22, 23]. If the degradation process is too fast, it may lead to swelling and focal inflammation. On the other hand, if it is too slow, the conduit could compress the nerve and cause chronic immune rejection.

## 4. Materials for the Making of Nerve Guidance Conduits

Depending on the original conduit material and manufacturing process, nerve conduits can be divided into biological and synthetic nerve conduits.

### 4.1. Biological Conduits

Biological conduits such as autologous arteries, veins, muscle, and isotype-variant or heterogeneous collagen tubes denatured skeletal muscle or muscle basal lamina [24, 25], human amniotic membrane [26], veins [27], and polyglycolic acid-collagen tubes [28]. Biomaterials such as vein, artery, muscle [29], and umbilical cord vessels have been widely used to repair relatively short nerve defects. These materials can provide support for the nerve in the short term and degrade to innocuous products after complete nerve regeneration. Some authors have used autogenetic epineurium [30, 31], normal nerve trunks [32], autogenic veins and autogenic small arteries, and even muscle fibers [33–37] to repair peripheral nerve injury and reported satisfying results.

### 4.2. Synthetic Nerve Conduits

They include nondegradable and degradable nerve conduits.

#### 4.2.1. Nondegradable Nerve Conduits: Silicone, Plastic, and Polytetrafluoroethylene Tubes

The silica gel canal was the earliest artificial conduit [38, 39]. Lundborg et al. [40, 41] used silicon tubes to repair nerve defects. Hollow silicon tubes have been used to repair less than 1 cm long nerve defects in rat sciatic nerve [42], and silicone tubes filled with SCs have been used to repair a 1.5 cm defect in rat sciatic nerve [43]. Although nondegradable nerve conduits eliminated the need to harvest autologous nerves, they always cause inflammation of the surrounding tissues and compression of nerve that could affect the regeneration of nerve axons [44]. Another disadvantage of those conduits is that they require a second surgery for removal, which could cause pain and more injury to the patient.

#### 4.2.2. Degradable Nerve Conduits

The commonly used degradable materials include collagen [45, 46], chitin [47–49], polyglycolic acid conduit, polylactic acid conduit, glycolide trimethylene carbonate conduit, polylactic acid conduit [50], polycarbolacton conduit, poly(lactide-co-glycolide)conduit, natural collagen, and hydrogel conduit.

Rosen et al. [51] compared autologous nerve graft alone and PGA plus type I collagen (extracellular matrix) grafting to bridge 5 mm defects in rat femoral nerve. After 11 months, autologous nerve graft was found superior to PGA grafting only by means of axonal diameter, but having no difference by means of axonal count or electrophysiologic or functional characteristics between the techniques. den Dunnen et al. [52] used poly (DL-lactide-epsilon-caprolactone) nerve guides and autologous nerve grafts to repair rat sciatic nerve defects. Application of biodegradable nerve conduits resulted in faster and qualitatively better nerve regeneration across a short nerve gap (1 cm) than with autologous nerve grafting method. Poly-3-hydroxybutyrate (PHB) nerve conduits were used to bridge long nerve defect (up to 4 cm) of rabbit common peroneal nerve and supported peripheral nerve regeneration up to 63 days and were proved to be suitable for bridging long nerve defects [53].

Researchers are enthusiastically investigating new biodegradable materials with excellent physical and chemical properties. Biodegradable chitosan-collagen and collagen tubes were proved to promote the growth of axons [54]. However, hollow biodegradable materials can be used to repair only relatively short nerve defects, and the functional recovery is still not satisfying. The combined use of fibronectin mats [55], allogeneic SCs [56, 57], ectogenous neurotrophic factors, and bridging tubes was proved to enhance neural regeneration after the injury [58]. PGA collagen tubes filled with collagen sponge and fibers infiltrated with laminae have repaired nerve defect of up to 8 cm in common peroneal nerves in dog [59]. This is the longest distance repaired by artificial nerve bridging so far.

## 5. Bioengineering of Conduits and Seeding with Support Cells

Tissue engineering techniques can be powerful modalities to improve the effectiveness of nerve conduit bridging. SCs have bioactivity and can produce nerve growth factors. Adherent molecules on the surface of SCs can secrete extracellular matrix and guide the growth of axons. Neurotrophic factors secreted by SCs may be the most important factors in the microenvironment for regenerating axons [60, 61]. SCs or stem cells with ordered scattering in tubes, similar to the bands of Bunger, may promote the growth of nerve axons. Nerve tubes with special 3D structure can include the regenerating axons and can mechanically guide axons [62]. Bioabsorbable and compound conduits (consisting of neurotrophic factors [63], nerve supporters [64, 65], SCs [66], and neural stem cells [67–69]) have promoted chemotactic regeneration of peripheral nerves and enhanced the effectiveness of nerve repair.

Gulati et al. [61] used cultured SC acellular grafts to repair 2 cm defects in rat peroneal nerve and found host axonal regeneration earlier and significantly better than hollow acellular grafts. Bunting et al. [70] introduced the use of bioabsorbable glass fibers with potential for the most challenging clinical cases that require bridging long interstump gaps. Sundback et al. [71] compared the use of poly(glycerol sebacate) (PGS) and poly(lactide-co-glycolide) PLGA by means of SC metabolic activity, attachment, proliferation, and apoptosis* in vitro* and found that PGS is an excellent candidate for neural reconstruction. Hadlock et al. [72] created polymer foam conduits with longitudinally aligned channels to implant SCs to provide a suitable environment for axonal regeneration. The polymer foam-processing method and unique channeled architecture allowed for controlled introduction of neurotrophic factors into the conduit. Fansa and Keilhoff [73] cultured isogenic SCs and implanted them into acellular autologous matrix: veins, muscles, nerves, and epineurium tubes. Good regeneration was noted in the muscle-SC group and impaired regeneration quality in the other groups (with or without SCs). The muscle-SC graft showed a systematic and organized regeneration, including a proper orientation of regenerating fibers. All venous and epineurium grafts showed more disorganized regeneration. Varejão et al. [74] compared functional peripheral nerve recovery in the rat sciatic nerve model after reconstruction of a 10 mm gap with a biodegradable poly(DLLA-epsilon-CL) nerve guide filled with fresh skeletal muscle or phosphate-buffered saline. Motor functional recovery was greater in the muscle-grafting group, with significant difference between 8 and 12 weeks. Axon regeneration progression was better with muscle-enriched tubes, especially from the distal nerve stump, than with hollow conduits.

## 6. Towards Use in Clinical Practice

In the last several decades, nerve conduits have been used in clinical practice and have significantly improved the functional recovery after peripheral nerve injury [75–78]. In the clinical trial of Chiu et al. [30], 22 patients with 34 nerve injuries were effectively repaired with autologous vein grafts as nerve conduits when selectively applied to bridge a small nerve gap (≤3 cm). Gu et al. [76] used chitosan/PGA nerve guidance channels to repair a 30 mm long median nerve defect in the right distal forearm of a 55-year-old male. Three years after the surgery, the patient showed satisfactory sensorial as well as functional recovery.

## 7. Future Prospects

Biodegradable nanomaterials are promising for manufacturing novel nerve conduits [79, 80]. Adequate density and 3D structure of scaffolds imbedded with SCs can lead to forming structures similar to bands of Bunger to enhance the regrowth of axons in peripheral nerve injury. To avoid immune rejection, SCs can be taken from umbilical stem cells or other tissues such as autologous adipose tissues. With the combined application of tissue, cell, and genetic engineering techniques, better functional recovery can be achieved after the peripheral nerve injury in the future.
